# A New Method for Characterization of Natural Zeolites and Organic Nanostructure Using Atomic Force Microscopy

**DOI:** 10.3390/nano2010079

**Published:** 2012-03-05

**Authors:** Domenico Fuoco

**Affiliations:** 1 Italian National Board of Chemists and Italian Chemical Society, Rome, 00187, Italy; Email: domenicofuoco@live.ca; 2 9260 Ukraine Street, Montreal, QC, H1R3W2, Canada

**Keywords:** atomic force microscopy (AFM), scanning electron microscopy (SEM), cetyltriammonium bromide (CTAB), zeolites, microtopography

## Abstract

In order to study and develop an economical solution to environmental pollution in water, a wide variety of materials have been investigated. Natural zeolites emerge from that research as the best in class of this category. Zeolites are natural materials which are relatively abundant and non biodegradable, economical and serve to perform processes of environmental remediation. This paper contains a full description of a new method to characterize the superficial properties of natural zeolites of exotic provenience (Caribbean Islets) with atomic force microscopy (AFM). AFM works with the simplicity of the optical microscope and the high resolution typical of a transmission electron microscope (TEM). If the sample is conductive, structural information of mesoporous material is obtained using scanning and transmission electron microscopy (SEM and TEM), otherwise the sample has to be processed through the grafitation technique, but this procedure induces errors of topography. Therefore, the existing AFM method, to observe zeolite powders, is made in a liquid cell-head scanner. This work confirms that it is possible to use an ambient air-head scanner to obtain a new kind of microtopography. Once optimized, this new method will allow investigation of organic micelles, a very soft nanostructure of cetyltriammonium bromide (CTAB), upon an inorganic surface such as natural zeolites. The data also demonstrated some correlation between SEM microphotographies and AFM 3D images.

## 1. Introduction

There are several natural substances which are not degradable and renewable, such as zeolites [1], that are the right solution to environmental remediation. In particular, some zeolites are ideal for aquifers polluted by toxic compounds such as hydrocarbons and organochlorinated compounds [2].

Zeolites (Greek, zein, “to boil”; lithos, “a stone”) are hydrated aluminosilicate minerals which have a micro-porous structure. The term was originally coined in the 18th century by a Swedish mineralogist named Axel Fredrik Cronstedt who observed, upon rapidly heating a natural mineral, that the stones begin to dance about as the water evaporated. Using the Greek words which mean “stone that boils”, he called this material zeolite. More than 150 zeolite types have been synthesized and 48 naturally occurring zeolites are known [1]. Zeolites have an “open” structure that can accommodate a wide variety of cations, such as Na^+^, K^+^, Ca^2+^, Mg^2+^ and others. These positive ions are rather loosely held and can readily be exchanged for others in a contact solution. Some of the more common mineral zeolites are: analcime, chabazite, heulandite, natrolite, phillipsite, and stilbite [1]. The general formula of a zeolite is

*M x/y* [(AlO_2_)*x*(SiO_2_)*y*] wH_2_O

**Figure 1:**
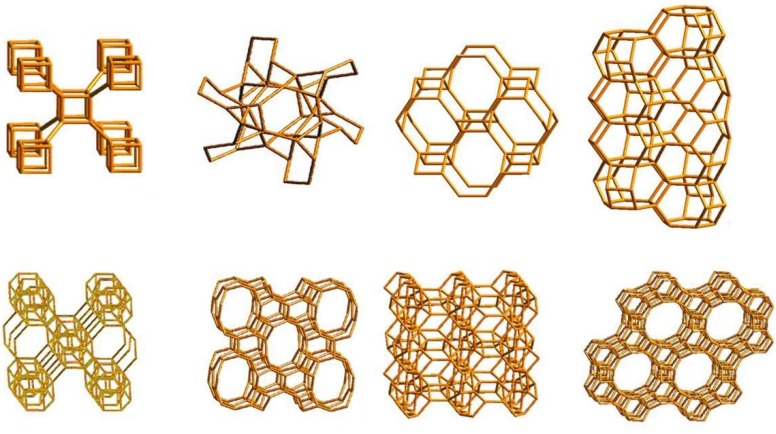
Molecular sieves. Microporous molecular structures of some Zeolites are shown into their atomistic representations. Image modified from Atlas of Zeolites.

*M* is the cation, w is the number of molecules of water and the ratio *x/y* is the dependent parameter of the structure between 1 and 5. Natural zeolites form where volcanic rocks and ash layers react with alkaline groundwater. Zeolites also crystallize in post-depositional environments over periods ranging from thousands to millions of years in shallow marine basins. Naturally occurring zeolites are rarely pure and are contaminated to varying degrees by other minerals, metals, quartz or other zeolites. For this reason, naturally occurring zeolites are excluded from many important commercial applications where uniformity and purity are essential. Zeolites are the aluminosilicate members of the family of microporous solids known as “molecular sieves” [3,4]. The term molecular sieve refers to a particular property of these materials, *i.e.*, the ability to selectively sort molecules based primarily on a size exclusion process (Figure 1). This is due to a very regular pore structure of molecular dimensions. The maximum size of the molecular or ionic species that can enter the pores of a zeolite is controlled by the diameters of the tunnels. These are conventionally defined by the ring size of the aperture, where, for example, the term “8 ring” refers to a closed loop that is built from 8 tetrahedrally coordinated silicon (or aluminium) atoms and 8 oxygen atoms. These rings are not always perfectly flat and symmetrical due to a variety of effects, including strain induced by the bonding between units that are needed to produce the overall structure, or coordination of some of the oxygen atoms of the rings to cations within the structure [5]. Therefore, the pore openings for all rings of one size are not identical [6,7].

## 2. Results and Discussion

The use of surfactant (or amphiphilic molecule)—referring to appropriate molecules such as ammonium salts of long chain (CTAB) [8]—is necessary to functionalize the surface of the samples in order to have a new sovramolecular structure which enables the exchange of anion [9,10]. The main idea is that individual chains of surfactant enter into the typical cavity of zeolite structures and other surfactants overlap the previous ones to form a classical fluid mosaic and/or micelles. The polar part, which contains the counter ion (usually a halide) is always turned outwards. This allows for the anions to be replaced by other ions present in the environment. The above phenomenon has already been theoretically predicted and observed with fluorescence studies [8]. In the present article a new method for the characterization of inorganic surfaces functionalized by amphiphilic organic molecules is discussed. The surfactant cetyltrimethylammonium bromide (CTAB) was chosen due to the fact that the literature demonstrates that it is already being used in work on zeolites, in reference to their curves of adsorption [11]. As expected from the packing parameters, CTAB gives rise to cylindrical micelle structures and with increases in concentration in hexagonal structures [12,13]. The different sovramolecular structures that result depend on the concentration of the surfactant and the temperature; at high temperatures the suvramolecular structures develop a *cloud point.* This cloud point corresponds to the temperature where the surfactant forms a precipitate. Once this point has been reached, it is no longer possible to identify more ordered sovramolecular structures. Starting out with our tablet of zeolite, we determined the reproducibility of our scanned image by observing the surface repeatedly and consistently. Once reproducibility had been established, we then coated the surface of our sample with “a drop” of solvent. The sample of zeolite was then further observed in order to determine any possibly occurring changes to the surface. In order to successfully determine the reproducibility of our measurements and dates, we had to establish the possibility of repeating the scan of the same area (that was coated with solvent).

**Figure 2:**
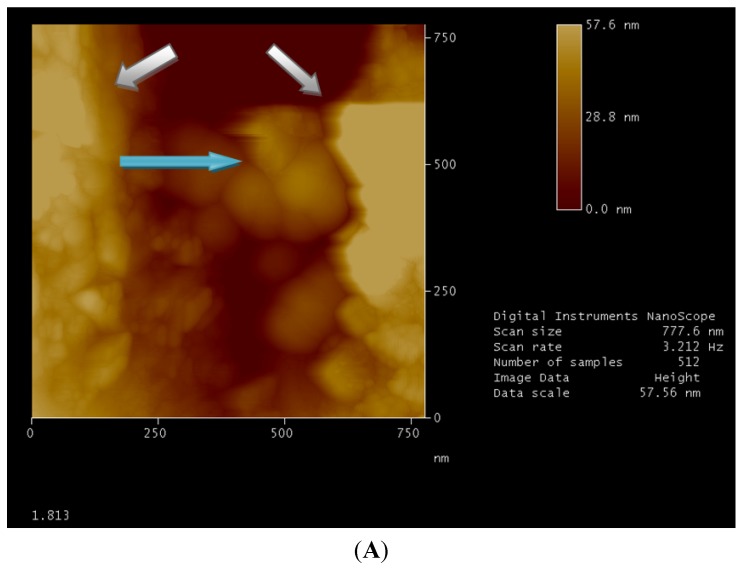

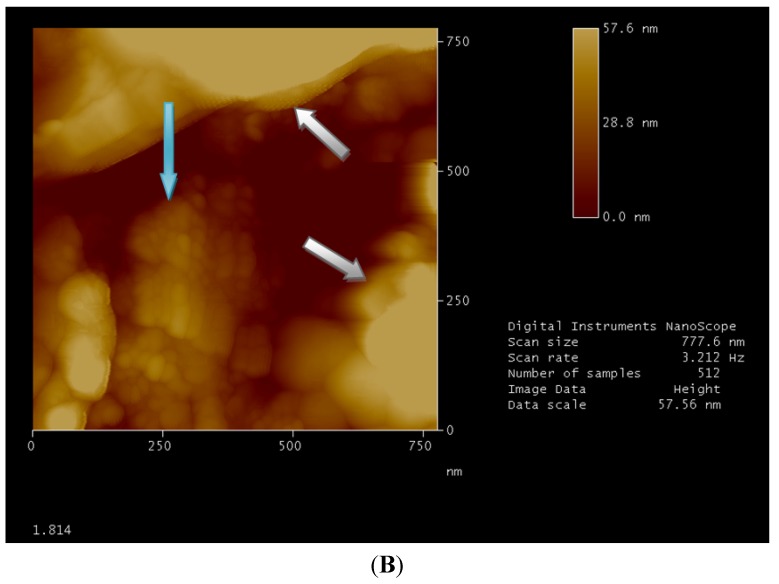
Atomic force microscopy (AFM) image obtained by “tapping mode”. Micellar structures are well clear into surface cavity with a medium diameter of a few tens of nanometers (blue arrow). Surface modifications, due to the solvent, build the walls of the cavity (white arrow). Image 1.813 (**A**) shows micellar structures with a medium diameter bigger than those in image 1.814 (**B**) which is attributable to *cloud point *concentration (as explained hereinabove).

**Figure 3:**
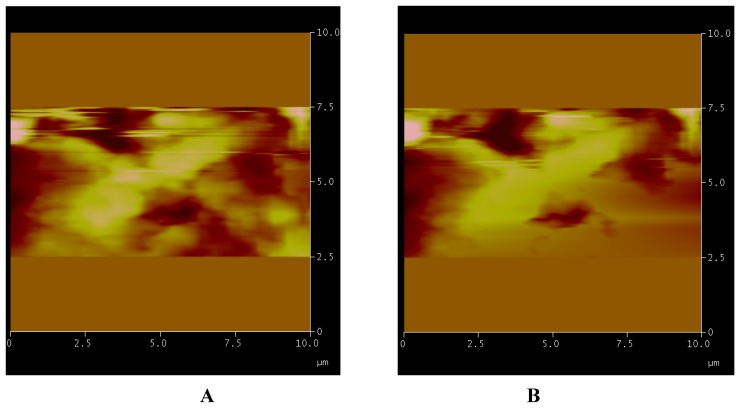

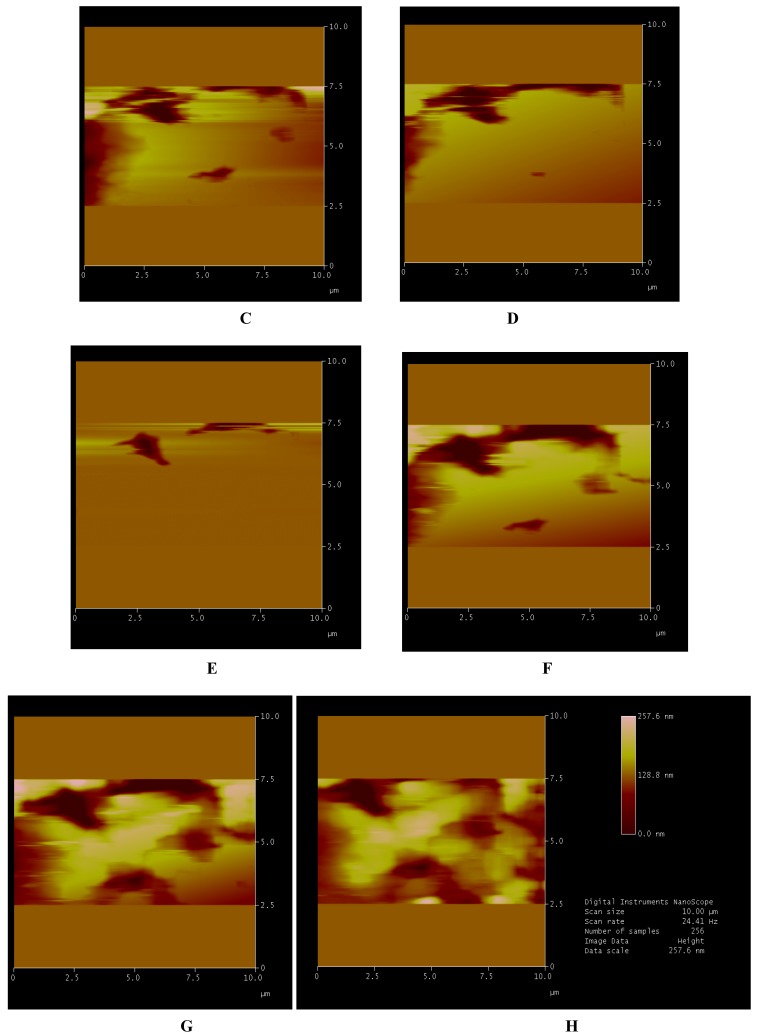
Solvent effect. The sequence of images shows the scanned area at full scale of 10 µm and its respective morphological structures (**A**); the advancing solvent front (**B**–**D**); the surface completely covered (**E**) and after evaporation (**F**). In **G** is shown the preservation of surface and its relative structures. In **H** is shown the picture with respective processed data during a single measurement.

(1) To allow the centering of an area of 200 nm from the side (of such size, *i.e*., that the images have a resolution of about 10 nm) procedures can “zoom” more gradually, becoming in succession areas (length side) respectively equal to 10 µm, 2.5 µm, 1 µm until 200 nm. Thus it has been possible to re-scan the same area, to the nearest ten nanometers (Figure 2).

(2) The images, obtained at different times, show that, without prejudice to possible easily identified artifacts, the details of tens of nanometers are faithfully reproduced. As this suggests, the technique used allows images with a real spatial resolution of tens of nanometers to be obtained. To observe the possible effects due to interaction of the tablet with the solvent alone, a volume of 50 µL of distilled water was placed on the surface, using a microvial for gascromatography, then left to spread over the region by capillarity. Several images were acquired without solution of continuity (this shows the versatility of the technique used). It is observed (Figure 3) that when the liquid spreads on the area being scanned, capillarity forces the same solvent to hide the structure of the surface; the effect of the solvent is that it seems to gradually cover the surface and temporarily creates a film of water molecules that prevents the acquisition of the data except at the deepest invaginations.

When the surface dries out, the overall look (scan length of 10 μm) is preserved, with clusters of spherical grains of size ranging from tens to hundreds of nanometers interspersed as well with areas of vacuum variable depth. These grains are often mixed together so that the image appears, at first view, less resolved. This phenomenon appears to be perfectly reproducible.

**Figure 4:**
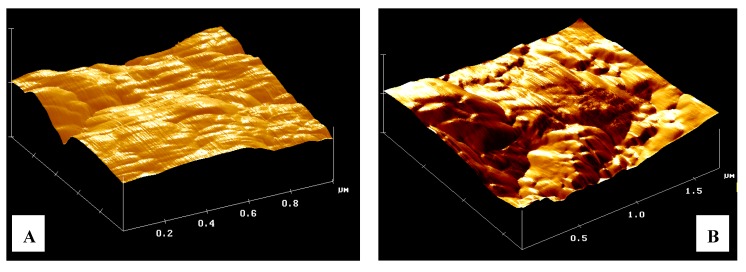

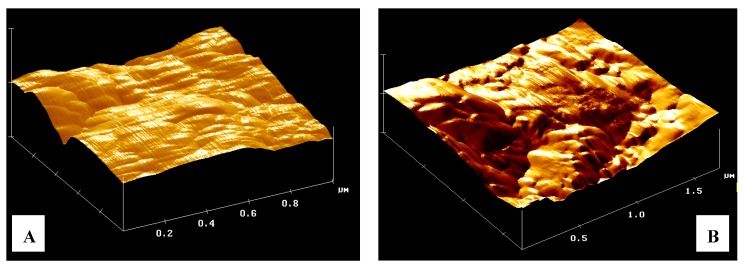
Three-Dimensional elaboration of scanned surface. The sample shows in the picture is compressed micronized powder of a zeolite derivative from a Clinoptilolite mineral. (**A**) surface at full scale of 1 µm; (**B**) same surface after the absorption of 150 µL of solvent; (**C**) same surface after the absorption of 150 µL of surfactant solution; (**D**) false color image of xy plane of same zeolite area.

In Figure 4A the data has a high resolution and low background noise and demonstrates how the surface at a scale of 1 micron is not flat Again, in Figure 4B, it is possible to see the effect of solvent on the zeolite, the structures which form the morphology after adsorption of water swell. Repeating the test with a surfactant solution, the picture obtained is presented in Figure 4C where the zeolite shows further invagination of the surface, which is directly proportional to the quantity of solution adsorbed. Echoing the acquisitions at different times (up to ten days) the same structures are found, demonstrating that the modeling of the surface is final and permanent over time. The false color image used to highlight the heights and depths on the lighter colors corresponds to the highest parts, and the darker colors represent the invaginations. In Figure 4D, an analysis, carried out by software, of the topography of a tablet of zeolites is reported. However, if we look at the images in greater detail, we note the presence of a pseudo-spherical shape with a diameter of several tens of nanometers. It is conceivable that these forms are really attributable to the surfactant micelles (Figure 2). Such presence is in fact spread over the whole area of the tablet by wet solution with surfactant.

*Sorption: Theoretical Limit between Ad/Absorption*

Sorption refers to the action of either absorption or adsorption. As such it is the effect of gases or liquids being incorporated into a material of a different state and adhering to the surface of another molecule. Absorption is the incorporation of a substance in one state into another of a different state (e.g., liquids being absorbed by a solid or gases being absorbed by a liquid). Adsorption is the physical adherence or bonding of ions and molecules onto the surface of another molecule. Observing an effect of sorption on a surface at nanoscale, it becomes particularly difficult to understand the nature of the phenomenon. Under these conditions, the forces of physical and chemical nature are the same [14,15].

Image Analysis with AFM and SEM Techniques

**Figure 5:**
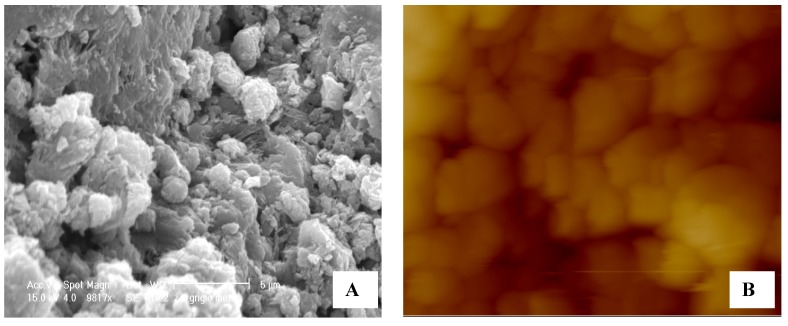

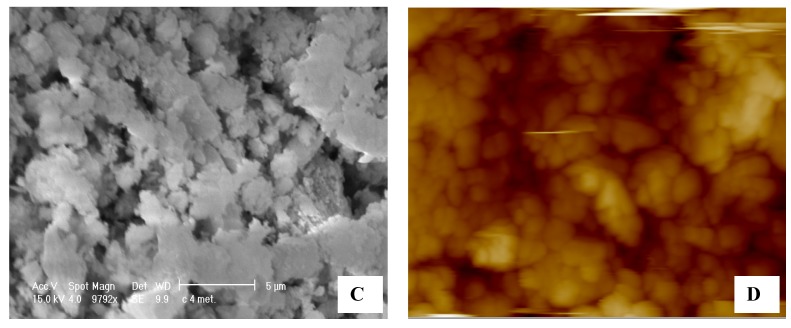
Comparison between AFM and scanning electron microscopy (SEM). **A** and **C** show a photomicrograph highlighting the presence of clusters of granules. In **B** and **D**, the AFM image of the same zeolites matrix powder. All the measures were carried out at the same full scale of 20 µm for better correlation.

This comparison is not intended to determine which instrument has the best image resolution because both are different techniques used for different purposes. It is evident from what has been said so far, that the AFM images can be helpful in understanding the SEM photomicrographs (Figure 5). However, an important aspect between the two microscopic techniques, is the need to work with a little conductive matrix using the “method of graphite” in the SEM observation. This practice alters deeply the surface of the mesoporous materials [16,17,18,19,20,21].

## 3. Experimental Section

### 3.1. Proprieties of Material

Natural zeolite studied in this work is a derivate of Clinoptilolite mineral (see mineral schedule, Table 1), coming from CUBA with ZOOK^®^ as its commercial name, obtained from Sereco Biotest, Perugia, Italy. Clinoptilolite is a natural zeolite comprising a microporous arrangement of silica and alumina tetrahedra. It has the complex formula: (Na,K,Ca)_2_–3Al_3_(Al, Si)_2_Si_13_O_36_12(H_2_O). It forms white to reddish tabular monoclinic tectosilicate crystals with a Mohs hardness of 3.5 to 4 and a specific gravity of 2.1 to 2.2. It commonly occurs as a devitrification product of volcanic glass shards in tuff and as vesicle fillings in basalts, andesites and rhyolites. It was described in 1969 after discovery in Owl Canyon, San Bernardino County, California. The use of clinoptilolite in industry and academia focuses on its ion exchange properties which have a strong exchange affinity for ammonia (NH_4_^+^) A typical example of this is in its use as an enzyme based urea sensor. It is also used as fertiliser. Research is generally focused around the shores of the Aegean Sea due to the abundance of natural clinoptilolite in easily accessible surface deposits.

**Table 1:** Characteristic of zeolite investigated in this work from the island of Cuba.

Mineralogical characteristics of Zook® Zeolites	Physics properties
Mineralogical composition (DXR): Clioptiolite 68%	Color: Green
Na_2_O	2.21		TiO_2_	0.45

### 3.2. Preparation of Samples

Understanding the impossibility to study natural crystals of zeolites using AFM, we must first powder them with mortar and pestle and then scour them with sieves of 40 μm mesh. Samples with two parallel surfaces were obtained using a press for normal tablets from powder. Parameters for a good compaction are 300 mg of powder under pressure of 10 tonnes for 30 seconds under high vacuum, and of course with inelastic powder.

### 3.3. Instruments

In several experiments, a surfactant solution was prepared by dissolving cetyltrimethyl ammonium bromide (CTAB) in water followed by 30 min of stirring to obtain the critical micellar concentration.

**Figure 6:**
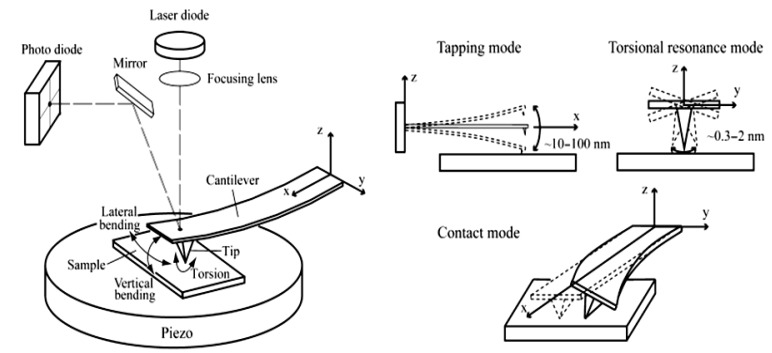
Atomic Force Microscopy. Schematic diagram of AFM and three different AFM operating modes. In this paper Tapping and Contact Modes are used.

AFM data were collected by a Digital Instruments-Veeco [22], now Bruker, MultiMode Nanoscope III (Figure 6). A silicon nitride cantilever (Digital Instruments) was scanned over the zeolite surfaces in contact and tapping mode at 8 lines/s. Imaging forces were minimized and estimated to be of the order of 5 nN. SEM data were performed in the Centro di Microscopia Elettronico of University of Perugia using a model Philips XL30. The high tension output can be linearly regulated from 0.2 to 30 kV and the magnification from 10 to 400,000°. This model is endowed with a LaB6 electron source that enables a very high definition of 27 Megapixel, coupled with a Silicon Graphics imaging processor.

### 3.4. Experimental Procedure

This work focuses on examining the changes induced on the surface of tablets using solvents and surfactant solutions and observing them by Atomic Force Microscopy (AFM). The experiment is carried out in four phases:

(1) Scanning some areas of the surface with different zoom from 10 μm until 200 nm as full scale.

(2) Adding 50 μL of distillated water (using a microvial for gascromatography) on the region scanned.

(3) After surface scanning, drying and again the same region from 10 μm until 200 nm as full scale, to observe the reproducibility of the phenomenon.

(4) When the phases above are set up, it is possible to observe organic nanostructure such as fatty acid micelles, using CTAB.

## 4. Conclusions

Zeolites are a class of microporous crystalline materials that have been widely used as catalysts, adsorbents and ion-exchangers. Zeolites contain uniformly sized pores in the range of 3–10 Å and can display molecular recognition, discrimination, and organization properties with a resolution of less than 1 Å. Zeolite characteristics depend on the nature of their pore openings and their hydrophobic or hydrophilic properties.

This article focuses on two issues: the development of a new method to investigate the surface properties of the zeolites with the AFM Nanoscope III and the study of changes induced on the surface of mesoporous matrices by surfactant solutions. The method discussed here is a novel and flexible strategy to use and is less expensive than other methods that study the microscopy of powder in solution with a special AFM liquid-cell mounted in the scanner head of the instruments.

AFM confirms its reliability as an advanced microscopy technique by multi-use: Working in contact mode the effect of solvent on a surface of mesoporous tablets of micronized zeolite is observed in real time. In contrast, working in tapping mode, well structured CTAB micelles can be observed. Finally, it was possible to observe the sovramolecular structures (micelles) of the surfactant and how they are arranged on the surface of the zeolite. All these images produced are important information to add to current knowledge on the possibilities for the use of AFM for these type of samples and for the characterization of powder of micronized materials. Future prospects are wide ranging, especially in the field of functionalized nanomaterials for environmental remediation using very economical systems.
